# MRI/Photoluminescence Dual-Modal Imaging Magnetic PLGA Nanocapsules for Theranostics

**DOI:** 10.3390/pharmaceutics12010016

**Published:** 2019-12-21

**Authors:** Yajie Zhang, Miguel García-Gabilondo, Anna Rosell, Anna Roig

**Affiliations:** 1 Institut de Ciència de Materials de Barcelona (ICMAB-CSIC), Campus UAB, 08193 Bellaterra, Catalonia, Spain; 2 Neurovascular Research Laboratory, Vall d’Hebron Institut de Recerca, Universitat Autònoma de Barcelona, 08035 Barcelona, Catalonia, Spain

**Keywords:** PLGA nanocapsules, magnetic resonance imaging, photoluminescence, drug delivery systems, magnetic targeting, multimodal imaging, theranostics

## Abstract

Developing multifunctional and biocompatible drug delivery nanoplatforms that integrate high drug loads and multiple imaging modalities avoiding cross-interferences is extremely challenging. Here we report on the successful chemical reaction of the high quantum yield biodegradable and photoluminescent polyester (BPLP) with the poly(lactic-co-glycolic acid) (PLGA) polymer to fabricate biocompatible photoluminescent nanocapsules (NCs). Furthermore, we transform the PLGA-BPLP NCs into a magnetic resonance (MR)/photoluminescence dual-modal imaging theranostic platform by incorporating superparamagnetic iron oxide nanoparticles (SPIONs) into the polymeric shell. In vitro phantoms confirmed the excellent MRI-r_2_ relaxivity values of the NCs whilst the cellular uptake of these NCs was clearly observed by fluorescence optical imaging. Besides, the NCs (mean size ~270 nm) were loaded with ~1 wt% of a model protein (BSA) and their PEGylation provided a more hydrophilic surface. The NCs show biocompatibility in vitro, as hCMEC/D3 endothelial cells viability was not affected for particle concentration up to 500 μg/mL. Interestingly, NCs decorated with SPIONs can be exploited for magnetic guiding and retention.

## 1. Introduction

Nanomedicine, which refers to the application of nanotechnology in medicine, offers valuable new tools for the diagnosis and treatment of many diseases. Nanoparticles are increasingly important by assisting to expedite the development of contrast agents, therapeutics, drug delivery vehicles, and theranostics in the context of nanomedicine [1]. Polymer-based nanoparticles are frequently proposed as drug carriers due to their biocompatibility and biodegradability, as well as the possibility of customizing their physicochemical properties for a specific drug or delivery route [2,3,4]. Among them, poly(lactic-co-glycolic acid) PLGA nanoparticles have gathered particular attention, since they encompass a number of interesting features: (i) FDA and European Medicine Agency approval in drug delivery systems for parenteral administration; (ii) well described formulations and methods of production adapted to various drugs i.e., hydrophilic or hydrophobic, small molecules, or macromolecules; (iii) protects the loaded drugs from degradation and possibility of sustained release; and, (iv) easy to modify to include targeted delivery or to provide tuned performance in a specific biological environment [5,6].

Theranostics is an emerging field that combines diagnostics and therapeutics into multifunction nanoparticle systems [7]. Drugs and nanocarriers in vitro/in vivo fate can be monitored while using noninvasive imaging techniques, such as magnetic resonance imaging (MRI) and fluorescent imaging, in order to optimize the route of delivery, biodistribution, and drug accumulation, among other factors [4,8,9]. PLGA nanoparticles can be used in diagnostic and therapeutic imaging by the addition of the imaging moieties during the particle synthesis. To date, large efforts have been devoted to conjugating PLGA with semiconducting quantum dots and organic dyes to create photoluminescent PLGA nanocarriers [9,10,11]. However, conventional approaches for physically blending imaging probes within the nanoparticles can lead to incomplete conclusions or misinterpretations on the nanoparticles’ biodistribution and fate [12,13]. Moreover, the inevitable photobleaching and low dye-to-polymer labeling ratios of organic dyes and the innate toxicity of quantum dots prevent their practical use in vivo [14,15]. Recently, a series of biodegradable photoluminescent (PL) polyesters (BPLPs) could derived inherently photoluminescent PLGA-BPLP copolymers with excellent biocompatibility, tunable luminescence and degradation rates, and good thermal and mechanical properties, thus expanding the biomedical applications of PLGA to highly desired optical imaging [16,17].

**Figure 1:**
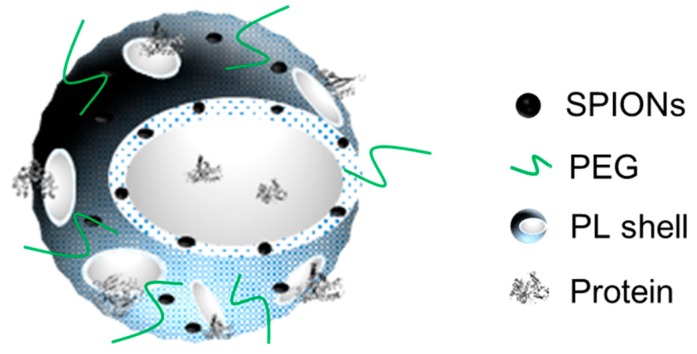
Schematic illustration of poly(lactic-co-glycolic acid)-biocompatible photoluminescent polymer nanocapsule (PLGA-BPLP NC) as a dual-modal imaging theranostic platform.

Here, we present PLGA nanocapsules (NCs) as a dual-modal imaging theranostic platform for magnetic targeting protein delivery (Figure 1). We report on the protocol for the fabrication of magnetic PLGA-BPLP NCs with intrinsic photoluminescence and MRI capacity being endowed by the incorporation of superparamagnetic iron oxide nanoparticles (SPIONs). Moreover, the NCs are functionalized with poly(ethylene glycol) (PEG), providing a hydrophilic surface that could result in an enhanced stealth effect. Unlike solid PLGA nanoparticles [4,18,19], in our NCs, the functional moieties are incorporated into the polymeric shell matrix to minimize interferences with the cargo being placed inside the NC; this is especially important for delicate payloads, such as proteins, enzymes, or microRNAs. In this study, bovine serum albumin (BSA) has been used as a model protein for evaluating the protein loading capability and release kinetics of the proposed nanocarrier.

## 2. Experimental Section

All of the reagents were purchased from Sigma-Aldrich (Merck KGaA, Darmstadt, Germany) unless specified otherwise.

### 2.1. Synthesis of PLGA-BPLP Copolymer

The PLGA-BPLP copolymer was synthesized by a slight modification of Yang’s method [16,17]. Step one involves the synthesis of a hydroxyl terminated BPLP pre-polymer. Briefly, citric acid, 1,8-octanediol, and l-cysteine with the molar ratio of 1:1:0.2 (5.76 g, 4.38 g, 0.72 g) were added into a flask of 50 mL with a stirring bar. Under a constant flow of argon, the reactants were melted by heating to 140 °C until a clear solution formed. The solution was allowed to react at this temperature for 80 min. and then stopped before the stirring bar stopped stirring completely in the increasingly viscous solution by adding 25 mL of 1,4-dioxane to dilute the produced prepolymer. The product was purified by dropwise precipitation from the 1,4-dioxane solution in water to remove the unreacted monomers. The final BPLP product was collected by centrifugation and then lyophilized.

In the second step, the PLGA-BPLP copolymer was synthesized while using the BPLP as a macroinitiator to react with l-lactide and glycolide via a ring-opening polymerization that was catalyzed by Tin (II) 2-ethylhexanoate (Sn(OCt)_2_). Briefly, l-lactide, glycolide and BPLP with the molar ratios of (75:25):1 or (50:50):1 were added into a reaction tube. Subsequently, Sn (OCt)_2_ (0.1 wt% of l-lactide and glycolide mixture) was added as a solution in dichloromethane which was then evaporated in vacuum. The tube was flushed with argon and capped and subsequently immersed in a 160 °C oil bath for 48 h. The obtained product was dissolved in chloroform and then purified by dropwise precipitation into pure ethanol to remove unreacted raw materials. Finally, the PLGA-BPLP copolymer was recovered by centrifugation and then dried in vacuum at room temperature. All of the PLGA-BPLP mentioned in the text were with molar ratios of (75:25):1, unless specified otherwise.

### 2.2. Synthesis of Oleic Acid Coated SPIONs

The SPIONs were synthesized by microwave assisted thermal decomposition in a microwave synthesizer (Discover SP, CEM Corporation, Matthews, NC, USA), and then coated by oleic acid (OA) [20]. The process to obtain homogeneous OA-SPIONs with an average diameter of 9 nm was as follows: 3.5 mmol of iron precursor Fe (acac)_3_ was dissolved in 4.5 mL of benzyl alcohol in a microwave reaction glass tube. Microwave irradiation was initiated at 60 °C for 5 min. to fully dissolve the precursor and, subsequently, the irradiation was kept at 210 °C for 30 min., and the reaction was then stopped and cooled down to room temperature. 4 mL of oleic acid in toluene (0.8 mmol/mL) was added immediately into the as-synthesized SPIONs dispersion followed by incubation under ultrasound for 1 h. Subsequently, the obtained OA-SPIONs were separated by centrifugation in five-fold of acetone. The pellet was redispersed in 4 mL of toluene in a glass vial and a magnet was attached on the wall for 5 s, the un-adsorbed suspension containing SPIONs with smaller size was discarded and the adsorbed pellet was redispersed in 6 mL of dichloromethane (DCM), followed by centrifugation at 4000 rpm for 5 min. to sediment the unstable big particles. Finally, the stable OA-SPIONs (9 nm) dispersion in DCM was centrifugated in five-fold of acetone and the pellet product was dried under vacuum and redispersed in DCM at the concentration required for use.

### 2.3. Fabrication of Functional PLGA-BPLP NCs and Encapsulation of BSA

Functional PLGA NCs encapsulating BSA were prepared by a double emulsion solvent evaporation method. Briefly, 50 μL of inner aqueous phase (W1) containing BSA (30 mg/mL) was emulsified in 500 μL of DCM organic phase (O) that was composed of 50 mg of different proportions of PLGA (RG502H, Mn 12,000)/PLGA-BPLP/PLGA-PEG (PLGA Mn 7000, PEG Mn 5000) and a certain amount of OA-SPIONs by sonication (VC505, Sonics & Materials Inc., Newtown, CT, USA) at 200 W for 28 s to form the first emulsion (W1/O). Afterwards, 2 mL of external aqueous phase (W2) with polyvinyl alcohol (PVA) (20 mg/mL) was added and the second emulsion (W1/O/W2) was formed by sonication for another 28 s. The temperature during the whole emulsion process was kept at 4 °C by using an ice bath. The resulting double emulsion was poured into 50 mL of MilliQ water and mechanically stirred at RT for 2 h to allow for complete evaporation of the organic solvent DCM and the formation of NCs. Finally, the NCs were washed three times with MilliQ water and lyophilized in 6 mL of trehalose aqueous solution (2 mg/mL). The as-obtained powder was stored at 4 °C with desiccant silica gel.

The non-PEGylated and PEGylated NCs have been labelled, as follows: NC1.non PEGylated (90 wt% PLGA-BPLP + 10 wt% PLGA) and NC2.PEGylated (90 wt% PLGA-BPLP + 3 wt% PLGA + 7 wt% PLGA-PEG), both containing ~6 wt% of SPIONs, as gathered in Table 1.

### 2.4. Physicochemical Characterization of PLGA-BPLP, SPIONs, and NCs

#### 2.4.1. Absorption Spectroscopies of the Polymers

Attenuated total reflectance-Fourier transform infrared (ATR-FTIR) characterization of the polymers was performed on a Bruker Vertex 70 FTIR spectrometer with a Pike Miracle Single-Bounce diamond crystal plate accessory at room temperature. The FTIR spectra were recorded over a wavelength range of 4000–500 cm^−1^ with a resolution of 4 cm^−1^. UV-Vis absorbance of the fluorescent polymers was recorded on a Varian Cary-5000 UV-Vis spectrophotometer while using a quartz cuvette with an optical path of 1 cm.

#### 2.4.2. Size Distribution and Disperse Stability of the NCs

Dynamic light scattering (DLS) (Zetasizer Nano ZS, Malvern Panalytical, Madrid, Spain) measurement of the hydrodynamic diameter and size distribution of NCs by intensity was performed by redispersing 0.5 mg of lyophilized NCs powder into 1 mL of MilliQ water.

Turbiscan (Turbiscan Lab, Formulaction, Toulouse, France) is used to detect the destabilization of the NCs suspension. 15 mL of 2 mg/mL NCs in the Turbiscan cell were scanned at all of the heights of the suspension with a time interval of 2 min. during 24 h. The back scattering signals at different heights of the cell were recorded and the delta of back scattering intensity was calculated by subtracting the reference time 0 s. The bottom part was defined as 1/5 of the liquid level.

Nanosight (NS300, Malvern Panalytical, Madrid, Spain) is used to measure the averaged size and concentration of the NCs water suspension. 0.2 mg/mL of NCs with 50 times dilution was pumped into the cell and the data were acquired and analyzed through the Nanoparticle Tracking Analysis (NTA) software along with the instrument.

#### 2.4.3. Electron Microscopies

Field emitting scanning electron microscope (SEM, FEI Quanta 200 FEG, Thermo Fisher Scientific, OR, USA) and transmission electron microscope (TEM, JEM-1210, JEOL Ltd., Tokyo, Japan) were used to study the morphologies of SPIONs and NCs. 0.5 mg of lyophilized powder was redispersed into 1 mL of MilliQ water and centrifuged at 4000 rpm for 10 min. for the SEM sample preparation of NCs. Subsequently, the supernatant was discarded to remove the trehalose (used for cryopreserving during lyophilization) and 1 mL of fresh water was added, the pellet of NCs was redispersed in water with ultrasound. Finally, 6 L of the slightly turbid suspension was deposited onto a small slice of silicon wafer stuck on the top of a carbon layer and dried at room temperature overnight. The sample was sputtered with Au-Pd (20 mA 2 min, Emitech K550, Quorum Technologies Ltd., East Sussex, UK). The TEM samples were prepared by placing and drying one drop of the corresponding NCs or SPIONs dispersion on a copper grid at room temperature.

#### 2.4.4. Magnetometry

Superconductive quantum interference device (SQUID, MPMS5XL, Quantum Design, San Diego, CA, USA) was used to measure the magnetization of NCs and SPIONs and calculate the SPIONs loading (wt%-SPIONs) of the magnetic NCs. Zero-field cooling and field cooling (ZFC-FC) measurement were used to determine the blocking temperature (T_B_) of the SPIONs. A gelatin capsule filled with about 7 mg of samples, together with some cotton wool, was inserted into the SQUID magnetometer sample holder and the hysteresis loop was measured from −50 kOe to 50 kOe. The saturation magnetization of the NCs (M_S_-NCs, emu/g) and of SPIONs (M_S_-SPIONs, emu/g) was used to calculate wt%-SPIONs, as follows:

$$\mathrm{wt}\%\text{-}\mathrm{SPIONs}=M_{S}\text{-}\mathrm{NCs}/M_{S}\text{-}\mathrm{SPIONs}\times 100\%$$

#### 2.4.5. Fluorescence Properties

Fluorescence spectra of the polymers and NCs were acquired on a spectrofluorometer (LS45, PerkinElmer Inc., Waltham, MA, USA). The excitation and emission slit widths were both set at 10 nm. The quantum yield of the polymers was measured by the Williams’ method [16]. Briefly, a series of BPLP/PLGA-BPLP solutions in the corresponding solvents were prepared with gradient concentrations. Maximal excitation wavelength was determined, which generated the highest emission intensity. The fluorescence spectra were collected for the series of solutions in the 10 mm fluorescence cuvette (Figure S1). The integrated fluorescence intensity, which is the area of the fluorescence spectrum, was calculated and then noted. Afterwards, the UV-Vis absorbance spectra were collected with the same solutions and the absorbance at the maximal excitation wavelength within the range of 0.01–0.1 Abs units was noted (Figure S1). The graphs of integrated fluorescence intensity vs. absorbance were plotted. The quantum yield was calculated according to the equation:

$$\Phi s=\Phi_{r}\left(\frac{{\mathrm{Slope}}_{s}}{{\mathrm{Slope}}_{r}}\right){\left(\frac{n_{s}}{n_{r}}\right)}^{2}$$

where, Φ = quantum yield; Slope = slope of the straight line obtained from the plot of intensity vs. absorbance; *n* = refractive index of the solvent; *s* = subscript denotes the sample; and, *r* = subscript denotes the reference used. Here, anthracene (Φ = 27% in ethanol when excited at 366 nm) was used as the reference.

The fluorescence intensities of different concentrations of NCs in water were quantified in a 96-well by a microplate reader (Spark, Tecan Group Ltd., Männedorf, Switzerland). The NCs were excited at the maximal excitation wavelength and the fluorescence signal was collected by area scan at the maximal emission wavelength.

Photostability was measured by continuously illuminating the polymer or NCs in a fluorescent confocal microscope (Leica SP5, Leica Microsistemas S.L.U., Barcelona, Spain) while using UV diode 405 nm excitation at different laser power (10%, 20%, 100%). The fluorescence images were acquired at a time interval of 1 s for 10 min., changes of mean fluorescence intensity of six region of interests (ROIs) in 10 min. were calculated while using software Las AF. The real laser power (W) at different percentage during 10 min. was monitored using a laser power meter.

### 2.5. MRI Phantoms of the NCs

In vitro agarose phantoms of NCs were prepared in Eppendorfs where a series of concentrations of NCs were vortexed and sonicated in agarose water solutions before the gel formed. The volume was kept at 1 mL with 0.63 wt% of agarose (Conda, Madrid, Spain). The corresponding iron doses (mmol/L) were calculated according to the wt% of SPIONs in each sample. T2 maps of the phantoms were acquired at 7 T in a 70/30 Bruker USR Biospec system (Bruker GmbH, Ettlingen, Germany), as follows: multi-slice multi-echo (MSME) sequence with echo time (TE) = 13 ms, repetition time (TR) = 4000 ms, field of view (FOV) = 5.5 × 11 mm, and three slices of 1 mm thickness. The quantitative T2 values were obtained from hand-drawn ROIs by using curve fitting in the Image Sequence Analysis (ISA) software along with the instrument.

### 2.6. In Vitro Toxicity Evaluation of the NCs

Two parallel methods assessed the toxicity of the NCs on human brain endothelial cells (hCMEC/D3): a viability assay based on WST-8 tetrazolium salt reduction (cell counting Kit-8, Dojindo) and direct cell counting. First, 10^4^ viable cells per well were seeded on a 24-well plate pre-treated with collagen (rat tail type I, Corning) in 400 μL of endothelial growth medium (EGM2 from Lonza with 2% fetal bovine serum and half the amount of the growth factors that were included in the kit). After incubation for 72 h at 37 °C with 5% of CO_2_, the cells were at 80–90% confluence and medium was changed to endothelial basal medium (EBM2, Lonza) containing NCs at 25, 50, 100, 500, and 1000 μg/mL. After 48 h, the cells were washed and incubated with 10% WST-8 solution for two hours. Culture supernatants were centrifuged at 20,000 rpm for 5 min. to remove NCs detritus that could interfere with the dye absorption before determining the absorbance at 450 nm. Cells were trypsinized and resuspended in growth medium and diluted 1:1 in Trypan Blue in order to perform cell counting in a Neubauer chamber. Cell viability and count are expressed as the percentage of absorbance or number of cells as compared with the control (vehicle without NCs). ANOVA test and Dunnett’s multiple comparisons post-hoc test was performed vs. the control.

### 2.7. In Vitro Observation of NCs Cellular Uptake

The hCMEC/D3 endothelial cells were seeded in cover-slips that were pre-treated with collagen (2 × 10^4^ cells/well in 24 well plates) and 24 h later were exposed to 50 μg/mL of empty fluorescent NCs. After an additional 24 h of culture, the wells were washed with PBS, fixated with 4% paraformaldehyde, and mounted with Vectashield antifade mounting medium (Vector Laboratories) with propidium iodide for nucleic acid counterstaining. Additionally, some of the cells were stained with PKH26 lipophilic fluorescent dye for cell membrane labeling according to the manufacturer’s protocol. Images at 63× were obtained on a fluorescent confocal microscope (LSM 980 with Airyscan 2 detector, Zeiss, Oberkochen, Germany).

### 2.8. BSA Loading in NCs and Release Kinetics

The albumin content of the NC was directly determined while using the CBQCA protein assay kit (Invitrogen™ ref. C6667), which determines the protein concentration based on the production of fluorescent products measurable at λ_ex_/λ_em_ = 450 nm/550 nm via non-covalent interaction between CBQCA and primary aliphatic amines of proteins. This highly sensitive fluorescence-based method showed compatibility with DMSO, SPIONs, detergents, and other substances that interfere with other commonly used protein determination methods. Lyophilized NCs encapsulating albumin as well as empty NCs as control were fully dissolved in DMSO at 100 mg/mL. The protein contents in the NCs lysates were measured and calculated based on the difference in fluorescence with the control and a calibration curve drawn with standard albumin solutions, the protein contents in the BSA solutions used for encapsulation were also measured. As listed in Table 1 for the two types of NCs, at least two replicate NCs batches of each were measured for the BSA loading and encapsulation efficiency (EE%) calculation. All of the measurements were performed in duplicate for each NCs batch. The experimental BSA loading in the NCs is expressed as μg of BSA per mg of NCs (μg/mg) or the wt% of the NCs, and the BSA EE% is calculated, as follows:

$$\mathrm{EE}\%\;\mathrm{BSA}=\frac{\mathrm{Experimental}\;\mathrm{BSA}\;\mathrm{loading}}{\mathrm{Nominal}\;\mathrm{BSA}\;\mathrm{loading}}\times 100\%$$

For the release studies, lyophilized NCs were resuspended in phosphate buffered saline (PBS) (pH 7.4, Sigma ref. D1408) at 10 mg/mL in low protein binding microcentrifuge tubes (Thermo Scientific© ref. 90410). NCs solutions were incubated at 37 °C in a vertical rotator for different time measures to simulate the in vivo environment: right after the resuspension (time 0), and after three hours, six h, one day, and seven days of incubation. In all cases, an aliquot of 200 μL was frozen at −80 °C until protein determination. Before the CBQCA assay, the aliquots were centrifuged at 15,000× *g* rcf to separate supernatant and pellet. The amount of protein release was directly calculated as total released protein and indirectly from the remaining protein in the pellet as indirect measure. For this purpose, the pellets were fully dissolved in DMSO and then compared with the intact NCs fully dissolved in DMSO (100 mg/mL) used as the 100% release set up. Four BSA-loaded NCs batches and one of H_2_O-NCs batch as control were used and measures done in duplicate. The release profiles were expressed in terms of cumulative release and plotted vs. time.

## 3. Results and Discussion

### 3.1. Photoluminescent PLGA-BPLP Copolymer

**Figure 2:**
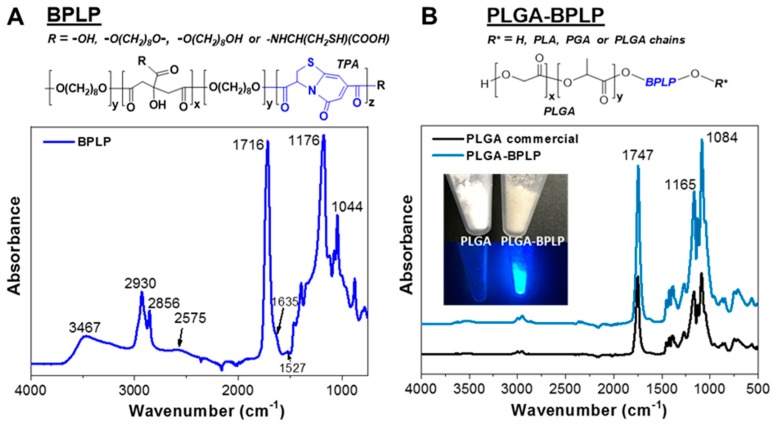
Attenuated total reflectance-Fourier transform infrared (ATR-FTIR) spectra of the as-synthesized biocompatible photoluminescent polymer (BPLP) and PLGA-BPLP copolymer confirming their chemical structures and the successful synthesis. (**A**) BPLP; (**B**) PLGA-BPLP and commercial PLGA as reference. Inset: fluorescence of the PLGA-BPLP under a UV lamp.

BPLPs are degradable oligomers synthesized from biocompatible monomers, including citric acid, aliphatic diols, and various amino acids via a convenient and cost-effective polycondensation reaction. BPLPs present some advantages over the traditional fluorescent organic dyes and quantum dots due to their cytocompatibility, minimal chronic inflammatory responses, controlled degradability, and excellent fluorescence properties [16]. Here, l-cysteine was selected and introduced into the polyester structure that was made of biocompatible monomers of citric acid and aliphatic 1,8-octanediol, since previously reported BPLP from this starting amino acid exhibited the highest quantum yield (62.3%) [16]. The fluorophore structure of BPLP was verified as a fused ring structure ((5-oxo-3,5-dihydro-thiazolopyridine-3,7-dicarboxylic acid, TPA) [21]. ATR-FTIR was also used to confirm the chemical structure of the as-synthesized BPLP (Figure 2A). Strong absorptions from the molecular backbone of the polyester were observed i.e., peaks at 1044 cm^−1^, 1176 cm^−1^, and 1716 cm^−1^ are attributed to the C=O stretch, C–O asymmetrical, and symmetrical stretches of the ester bond, respectively, the peaks at 2930 cm^−1^ and 2856 cm^−1^ are attributed to the C-H stretches of alkane from 1,8-octanediol and the band near 3467 cm^−1^ is from the −OH. NH bending of the secondary amide at 1527 cm^−1^ and −SH at 2575 cm^−1^ confirm that l-cysteine is chemically bound to the poly(diol citrate) chain. The shoulder band near 1635 cm^−1^ is attributed to the C=O stretching of the tertiary amide from the TPA ring. The average molecular weight (M_w_) of BPLP measured by matrix-assisted laser desorption/ionization time of flight mass spectroscopy (MALDI-TOF-MS) was 1044 g/mol (Figure S2). The BPLP oligomer served as a macroinitiator to react with l-lactide and glycolide via a ring-opening polymerization to produce PLGA-BPLP [17]. The as-synthesized PLGA-BPLP (75:25):1 with molar ratios equal to 75:25 for l-lactide to glycolide and equal to 1:100 for BPLP to total l-lactide and glycolide was reported to have desirable glass transition temperature (Tg, 32.5 °C), mechanical properties, fluorescence properties, and degradation rate of the resulting product [17]. Moreover, we have proved here that the 75:25 formulations are more suitable for the fabrication of NCs by a double mini-emulsion method than the PLGA-BPLP (50:50):1 one, as shown in the following section.

The obtained PLGA-BPLP copolymer (Figure 2B inset) exhibits the inherent photoluminescence from the BPLP. The similarities of bands and shapes of the IR spectra of the as-synthesized PLGA-BPLP and commercial PLGA (Figure 2B), for instance, bands at 1084 cm^−1^, 1165cm^−1^, and 1747 cm^−1^ from the ester bonds in PLGA indicate their similar chemical structure, given the fact that BPLP is a very small portion of the PLGA-BPLP copolymer.

**Figure 3:**
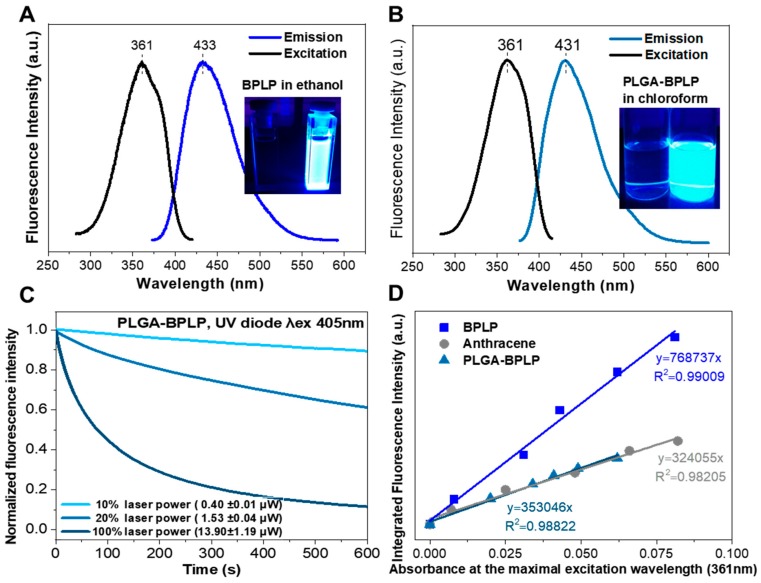
(**A**,**B**) excitation and emission spectra of the as-synthesized BPLP and PLGA-BPLP. Insets: fluorescence of BPLP and PLGA-BPLP dispersed in solutions under a UV lamp; (**C**) photostability evaluation of PLGA-BPLP powder under confocal microscope at different laser power, fluorescence intensity expressed as the percentage vs. the value at the initial time; and, (**D**) fluorescence intensity-absorbance curves of BPLP, PLGA-BPLP, and anthracene used as a reference used to calculate quantum yields.

The fluorescence of the as-synthesized BPLP and PLGA-BPLP was evaluated and Figure 3A,B depict the excitation and emission spectra. The similar spectra further confirm the inherent photoluminescence of PLGA-BPLP from BPLP. Importantly, the fluorescence intensity of PLGA-BPLP only slightly decreased (10%) after 10 min. of continuous illumination under confocal microscope at 10% of laser power (0.40 ± 0.01 µW) (Figure 3C). When considering the laser power applied to observe stained cells is generally less than 10%, our photoluminescent polymer would exhibit good photostability at in vitro conditions. The calculated high quantum yields of BPLP (64%) and PLGA-BPLP (33%) from Figure 3D are consistent with the previously reported values [16,17]. The remarkable fluorescence properties that are shown here endow the PLGA-BPLP copolymer with high potential for the fabrication of functional photoluminescent NCs.

### 3.2. Fabrication of PLGA-BPLP NCs Combining Other Functional Moieties

**Figure 4:**
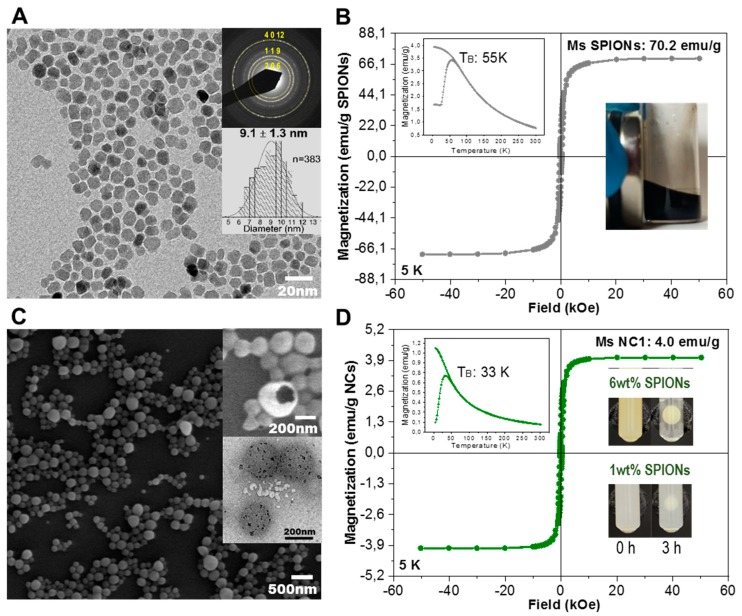
(**A**) Representative transmission electron microscope (TEM) image of the oleic acid (OA) coated SPIONs with the upper inset showing the selected area electron diffraction (SAED) pattern of maghemite and lower inset a size distribution histogram of the particles in the image; (**B**) hysteresis loop (5 K) and ZFC-FC (inset) for the OA-superparamagnetic iron oxide nanoparticles (OA-SPIONs), inserted picture shows the stable OA-SPIONs in dichloromethane attracted by an external magnet; (**C**) representative SEM image of lyophilized NCs with the upper inset showing the hollow core of a nanocapsule and lower inset a TEM image of three NCs with the SPIONs visible as black spots well distributed in the polymer matrix; (**D**) hysteresis loop (5 K) and ZFC-FC (inset) for the lyophilized nanocapsule batch NC1, inserted pictures show the water suspension of these NCs (2 mg/mL) where the 6 wt% loading of SPIONs were adsorbed faster to the tube wall on the magnet (diameter 8mm, surface field ~0.4 T) side than that of 1 wt% loading.

PLGA-based NCs with BSA encapsulated were prepared by a double emulsion solvent evaporation method with slight modifications from our previously reported method [22]. Functional moieties, such as PLGA-BPLP for the fluorescence imaging, PLGA-PEG to increase hydrophilicity and sealthness, and SPIONs for magnetic targeting and MRI (Figure 4A,B) can be incorporated to the organic phase during the NCs fabrication process (see Table 1). The first important remark is that NC morphology was not affected, even when using 100 wt% of PLGA-BPLP (75:25):1 (Figure S3A). A formulation with 90 wt% of PLGA-BPLP (75:25):1 was selected to allow for NC PEGylation by the addition of 7 wt% of PLGA-PEG and for the benefit of higher fluorescence intensity (Figure S4). Note that 7 wt% of PLGA-PEG (3 wt% PEG, Figure S3B) (NC2) was found to be the maximum amount that can be mixed in the organic phase during the NCs fabrication process, due to the amphiphilic property of PLGA-PEG. For a larger wt% of PLGA-PEG, the morphology and size of NCs were not maintained. As shown in the SEM image (Figure 4C), non-PEGylated and PEGylated NCs, as listed in Table 1, both contain ~6 wt% of SPIONs and depict homogeneous spherical morphologies and sizes (d.nm ~270) being similar to other reported PLGA systems that are suitable for intravenous administration [23]. The upper inset in Figure 4C shows a representative broken NC, exposing the hollow core where the protein drug is loaded. We have also found that the PLGA-BPLP (50:50):1 polymer from using initial molar ratio of LA:GA = 50:50 and BPLP:(LA + GA) = 1:100 was not as suitable for the fabrication of NCs. NCs with homogeneous morphology and narrow size distribution were attained only up to a maximum of 30 wt% of modified PLGA (Figure S3C) and without the possibility of further adding PLGA-PEG when using PLGA-BPLP (50:50):1 (Figure S3D). These results are in accordance with the higher glass transition temperature and better mechanical properties of PLGA-BPLP (75:25):1 over those of PLGA-BPLP (50:50):1 [17]. As expected, the NCs with a higher fraction of PLGA-BPLP (75:25):1 (90 wt%) show higher fluorescence intensity than the ones that were obtained with PLGA-BPLP (50:50):1 (30 wt%) at the same concentration (Figure S4).

Regarding magnetic loading, up to 6 wt% of SPIONs could be loaded without affecting the NCs morphology and yielding a saturation magnetization (M_S_) value of around 4 emu/g NCs (Figure 4D). In addition, the lower blocking temperature (T_B_, 33 K) of SPIONs in the NCs (Figure 4D inset) than the SPIONs of dry powder (55 K, Figure 4B inset) further demonstrates that the SPIONs are well dispersed in the polymer matrix. Note that a high magnetic loading is desirable for the magnetic retention of NCs. This is illustrated in the inset images of Figure 4D, where the darker-coloured water suspension of NCs with 6 wt% of SPIONs were adsorbed faster to the tube wall on the magnet side than the NCs with 1 wt% SPIONs loading at the same concentration, promisingly benefiting the magnetically targeted drug delivery as compared to the previously reported results [4,24]. Note that the superparamagnetic behaviour of NCs at room temperature (lack of coercivity, Figure S5) ensures no magnetic interactions among NCs in the absence of an external magnetic field, minimizing the risk of embolization during i.v. administration.

**Figure 5:**
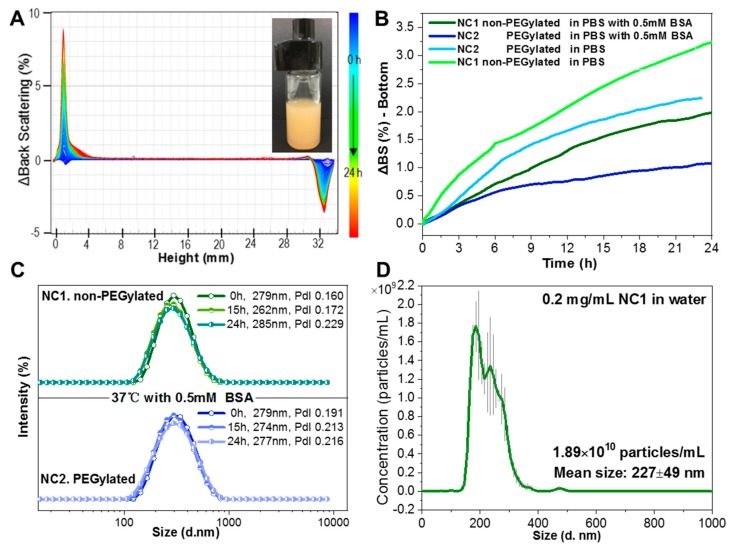
(**A**) Back scattering intensity change of the NC2 PBS suspension (shown inset) containing 0.5 mM of BSA at different height of the vial along 24 h measured by Turbiscan; (**B**) quantified back scattering intensity change of the bottom part of the vial along 24 h for NCs suspension in different media measured by Turbiscan; (**C**) dynamic light scattering (DLS) size distributions of NC1 and NC2 PBS suspensions with 0.5 mM of BSA along 24 h; (**D**) quantitative number concentration and size distribution of the nanocapsules measured by Nanosight (n = 3, mean ± SD with error bar).

It is well reported that the surface PEGylation of engineered nanoparticles provides them with stealth character increasing blood circulation time since nanoparticles are less visible to the reticulo-endothelial system [6]. We evaluated the flocculation regime of the NCs with a Turbiscan to confirm successful surface modification with PEG, since the long hydrophilic PEG chains (Mn 5000) on the surface of the NCs are expected to increase the stability of the NCs in water suspension and decrease the sedimentation rate. Sedimentation of the NCs suspension was monitored for 24 h. Figure 5A shows that, as NCs sedimentation progresses, the back scattering signal of the bottom part of the suspension increases from an increasingly higher concentration of NCs, while the signal of the top part decreases. The sedimentation rates of non-PEGylated and PEGylated NCs were compared with or without a physiological concentration of BSA (0.5 mM) in Figure 5B, as expected the bottom back scattering signal of the PEGylated NCs media increases at a slower rate than the non-PEGylated ones both with and without BSA, which demonstrates a better dispersibility of the NCs due to the surface hydrophilic PEG chains. Note that the sedimentation rates of non-PEGylated and PEGylated NCs both slow down with the physiological concentration of BSA probably due to the interaction of NCs with the dense BSA solution. Additionally, in Figure 5A, the back scattering signal of the middle part did not vary with time, which means that the NCs were monodispersed at the physiological concentration of BSA and flocculation or coalescence did not occur during the 24 h period. This is in consistent with the DLS size distribution results that are shown in Figure 5C, both non-PEGylated and PEGylated NCs remained monodisperse at the physiological concentration of BSA, which is of great advantage for the i.v. administration and in vivo blood circulation. Nanosight was also used as an additional technique for the determination of size and the concentration of the NCs (Figure 5D). The results show a similar size distribution as obtained by DLS. From the number concentration of the NCs, we can determine a mean mass of 1.06 × 10^−11^ mg/NC.

### 3.3. Imaging Performance of the Magnetic Photoluminescent NCs

**Figure 6:**
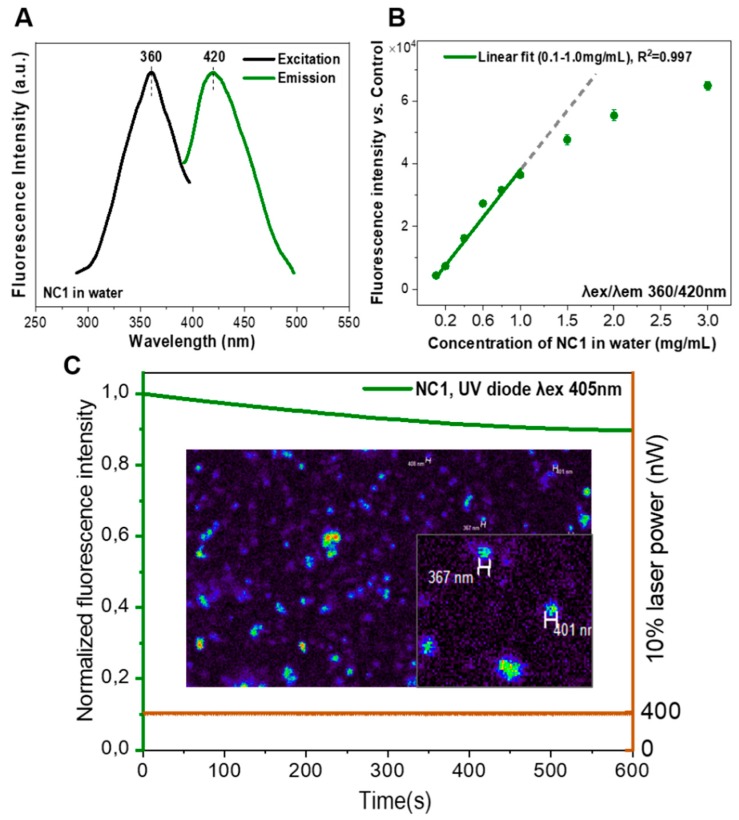
(**A**) Excitation and emission spectra of the nanocapsules (NCs) water suspension; (**B**) fluorescence intensity of different concentrations of NCs measured by microplate reader (n = 2, values represent mean ± sd and subtract values of control non-fluorescence NCs); and, (**C**) photostability evaluation of NCs under confocal microscope at 10% laser power, fluorescence intensity expressed as the percentage vs. the value at the initial time, inset: NC1 water suspension observed at 60× lens.

The fluorescence of the NCs was evaluated, and Figure 6A depicts the excitation and emission spectra. The spectra are similar to those of the PLGA-BPLP polymer shown in Figure 3B. Importantly, the incorporation of SPIONs in the polymer matrix does not quench the fluorescence of NCs. Note that a small displacement of the emission peak wavelength was observed for aqueous dispersed fluorescent NCs when compared to the emission peak of the polymer in a chloroform solution (Figure 3B), which we ascribe to the different interaction of the fluorescent probe with the two solvents. The fluorescence intensities of NCs show a linear dependency on the NCs concentration within a range of 0.1 to 1.0 mg/mL; at higher concentrations the fluorescence shows a trend towards saturation (Figure 6B). NCs can be clearly imaged with a fluorescence confocal microscope and they show a very good photostability while using 10% of laser power, which is ideal for the observation of in vitro cellular uptake (Figure 6C). The strategy used here confers intrinsic photoluminescence to the PLGA NCs without introducing any cytotoxic quantum dots or photo-bleaching organic dyes when compared to other more conventional approaches that physically blend imaging probes within the carrier that can lead to misinterpretations on the tracing of the carrier [12,13], which may greatly expand the applications of this drug carrier.

**Figure 7:**
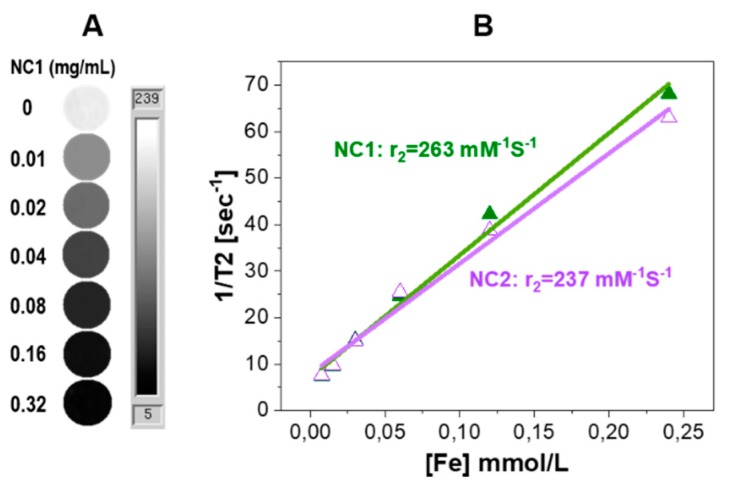
(**A**) T2 maps of a series of concentrations of NC1 in agarose phantoms; (**B**) r_2_ relaxivity evaluation for the NCs.

Phantom studies were conducted to confirm the MRI performance of the capsules. Phantoms of NCs that were dispersed in agarose gel at various concentrations were prepared (Figure 7A). Spin-spin relaxation time (T2) maps clearly exhibit signal decay in a concentration dependent manner. The calculated transverse relaxivity (r_2_) values at 7 Tesla of both non-PEGylated NC1 (263 mM^−1^s^−1^) and PEGylated NC2 (237 mM^−1^s^−1^) are similar as those seen in Figure 7B, as expected, further demonstrating the similar loading and distribution of SPIONs in the polymer shell matrix for both systems. When compared with other clinically used SPIONs systems, such as Feridex (98 mM^−1^s^−1^) and Resovist (151 mM^−1^s^−1^) [25], the much higher r_2_ value of our NCs formula is expected to be useful for in vivo MRI tracking of the NCs.

### 3.4. Cell Viability after NCs Uptake

**Figure 8:**
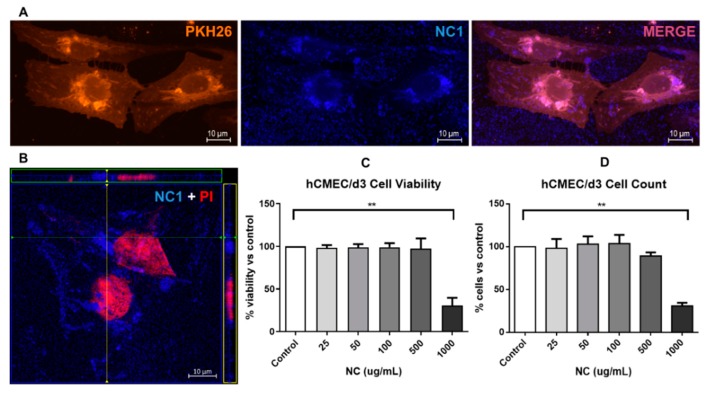
NCs uptake and cytotoxicity in human brain endothelial cells. (**A**) Representative images of hCEMC/d3 cells stained with membrane dye PHK26 and exposed to 50 μg/mL of NC1 for 24 h (63× magnification); (**B**) Orthogonal view of a Z-stack of Propidium Iodide (PI) stained cells (showing the cell nuclei) and the fluorescent NCs; (**C**,**D**) hCEMC/d3 cells were treated for 48 h with different concentrations of NC1 and cell viability was determined with WST-8 reduction assay or tripsinized and counted in a Neubauer chamber, (n = 3–4, values represent mean ± SEM, ** *p* < 0.01).

Photoluminescent NCs were incorporated by brain endothelial cells after several hours in culture, as seen in Figure 8A, with cytoplasmic localization of the NCs in perinuclear structures compatible with Golgi bodies and endosomes. This subcellular localization was confirmed by Z-stack images (Figure 8B). Importantly, this cellular uptake was biocompatible for endothelial cells, as the main exposed cells during NCs circulation in blood vessels, since viability tests did not show signs of cell toxicity at a wide range of NCs concentrations up to 500 μg/mL (Figure 8C,D) and 48 h exposure. Only extremely high doses (1000 μg/mL) with noticeable occupying space difficulties for cell culturing showed a significant reduction in cell viability and number.

### 3.5. Protein Loading and In Vitro Release

Protein loading and encapsulation efficiency were determined by lysing NCs with DMSO and measuring total protein content. The BSA loading content was determined as ~10 µg BSA/mg NCs (1 wt%) with an EE% of around 40% for both NC2.PEGylated and NC1.non-PEGylated systems, as listed in Table 1, which indicated that the incorporation of PEG does not affect the protein encapsulation process. Note that a protein loading of 1 wt% is much higher than other reported values (0.03 wt% of vessel endothelial growth factor (VEGF) loaded PLGA NCs) [22] and the EE% of 40% is comparable to the PLGA NCs loaded with neurotrophin-3 or brain-derived neurotrophic factor (47%) [26].

**Figure 9:**
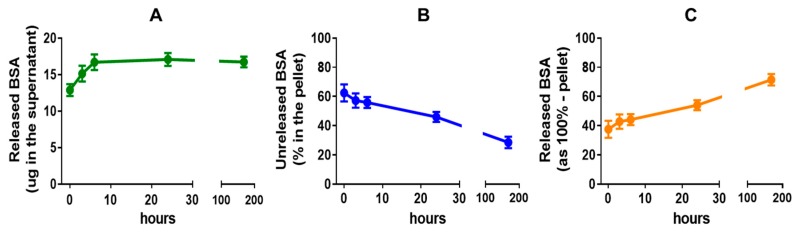
Protein release temporal profile of the NCs. (**A**–**C**) NC1 in PBS (10 mg/mL) were incubated at 37 °C in rotation and the released BSA quantified as total mass of protein released (**A**) together with BSA content in the remaining NCs pellet by DMSO lysis (**B**) and the released BSA calculated indirectly from the pellet values (**C**). The percentage was calculated versus intact unreleased NCs lysated also in DMSO (n = 8, values represent mean ± SEM).

BSA-loaded NCs were able to release protein cargo over time at physiological temperature in PBS media (32% protein release in one week). Figure 9A shows a fast BSA release within the first hours, but not after one day, which could be related to the protein degradation in ex vivo conditions of our assays. The amount of released protein in one week was similar when indirectly measured from the pellet retained protein (Figure 9B,C), although the release profile showed a more sustained pattern over time, which could be associated to the protein that was trapped within the PLGA polymer.

By using the model protein BSA, here we were able to prove the loading capacity and release kinetics of the drug carrier, yet the preservation of protein functionality after the encapsulation process still needs to be investigated. Nevertheless, in a previous work, we have shown that VEGF could be encapsulated following a similar route and VEGF effect on cell proliferation could be determined [22], which implied the preserved protein functionality.

## 4. Conclusions

We here transform PLGA nanocapsules (NCs) into a highly sensitive, MRI/photoluminescence dual-modal imaging theranostic platform for drug delivery by integrating the biocompatible and photoluminescent polyester BPLP into the PLGA molecular structure, as well as by incorporating superparamagnetic iron oxide nanoparticles (SPIONs). Furthermore, we have shown that PEGylation provides a hydrophilic surface to the NCs slowing down their flocculation rated and without modiying the size, SPIONs content or protein loading capacity of the NCs. In all cases, the functional moieties are embedded in the PLGA shell with minimal interferences between them or with the therapeutic protein. The developed magnetic PLGA-BPLP NCs show biocompatibility in vitro. In this regard, the NCs did not affect the viability of endothelial cells in culture for concentration up to 500 μg/mL and 48 h incubation. Finally, we have shown that the NCs can contain 1 wt% of protein in their core achieved at fabrication level and that one third of the encapsulated protein is released in the first week. Interestingly, the NCs decorated with SPIONs can be exploited for magnetic retention and magnetic guiding.

## Figures and Tables

**Table 1:** Summary of the two main formulations for the NCs. Oleic acid coated-SPIONs and PLGA-BPLP and PLGA-PEG were mixed in the organic phase during the double miniemulsion process.

NCs Type	Shell Polymers (wt%)			SPIONs Loading (wt%)	M_s_ (eum/g)	Size (DLS)		BSA Loading (wt%)	BSA EE%
	PLGA	PLGA-BPLP *	PLGA-PEG			d. nm	PdI		
NC1.non-PEGylated	10	90	**/**	5.7	4.0	272	0.11	0.96	38.5
NC2.PEGylated	3	90	7	6.0	4.2	265	0.05	1.01	41.3

* PLGA-BPLP from initial molar ratios (LA:GA):BPLP = (75:25):1.

## Supplementary Materials

The following are available online at https://www.mdpi.com/1999-4923/12/1/16/s1. Figure S1: UV-Vis absorbance spectra and fluorescence spectra of the as-synthesized A/B) BPLP, C/D) PLGA-BPLP and E/F) reference anthracene at a series concentrations for fluorescence quantum yield calculation, Figure S2: Matrix-assisted laser desorption/ionization time of flight mass spectroscopy (MALDI-TOF-MS) was used to determine the average molecular weight (Mw) of BPLP. Average Mw calculated from the mass spectrum of BPLP is 1044 g/mol. For the analysis, the sample was spotted in a MALDI plate with 1,8,9-Antracenotriol (Ditranol) and analyzed using a mass spectrometer (Bruker Daltonics Ultraflex TOF/TOF) in reflectron mode, Figure S3: SEM images and DLS size distributions of NCs fabricated by different types of PLGA-BPLP, Figure S4: Fluorescence intensity of different concentrations of NCs measured by microplate reader at the maximal emission wavelengths (values have subtracted values of control non-fluorescence NCs), Figure S5: Hysteresis loop (300 K) and ZFC-FC (inset) measurements of the lyophilized nanocapsule batch NC2.
