# Supplementary material for: MRI/Photoluminescence Dual-Modal Imaging Magnetic PLGA Nanocapsules for Theranostics
Source: Pharmaceutics. 2019 Dec 21;12(1):16. doi: 10.3390/pharmaceutics12010016 (PMC7023350; doi:10.3390/pharmaceutics12010016)
Supplement: Supplementary file 1 [file pharmaceutics-12-00016-s001.pdf]

pharmaceutics

Supplementary Materials: MRI/Photoluminescence Dual-Modal Imaging Magnetic PLGA Nanocapsules for Theranostics

Yajie Zhang, Miguel García-Gabilondo, Anna Rosell and Anna Roig

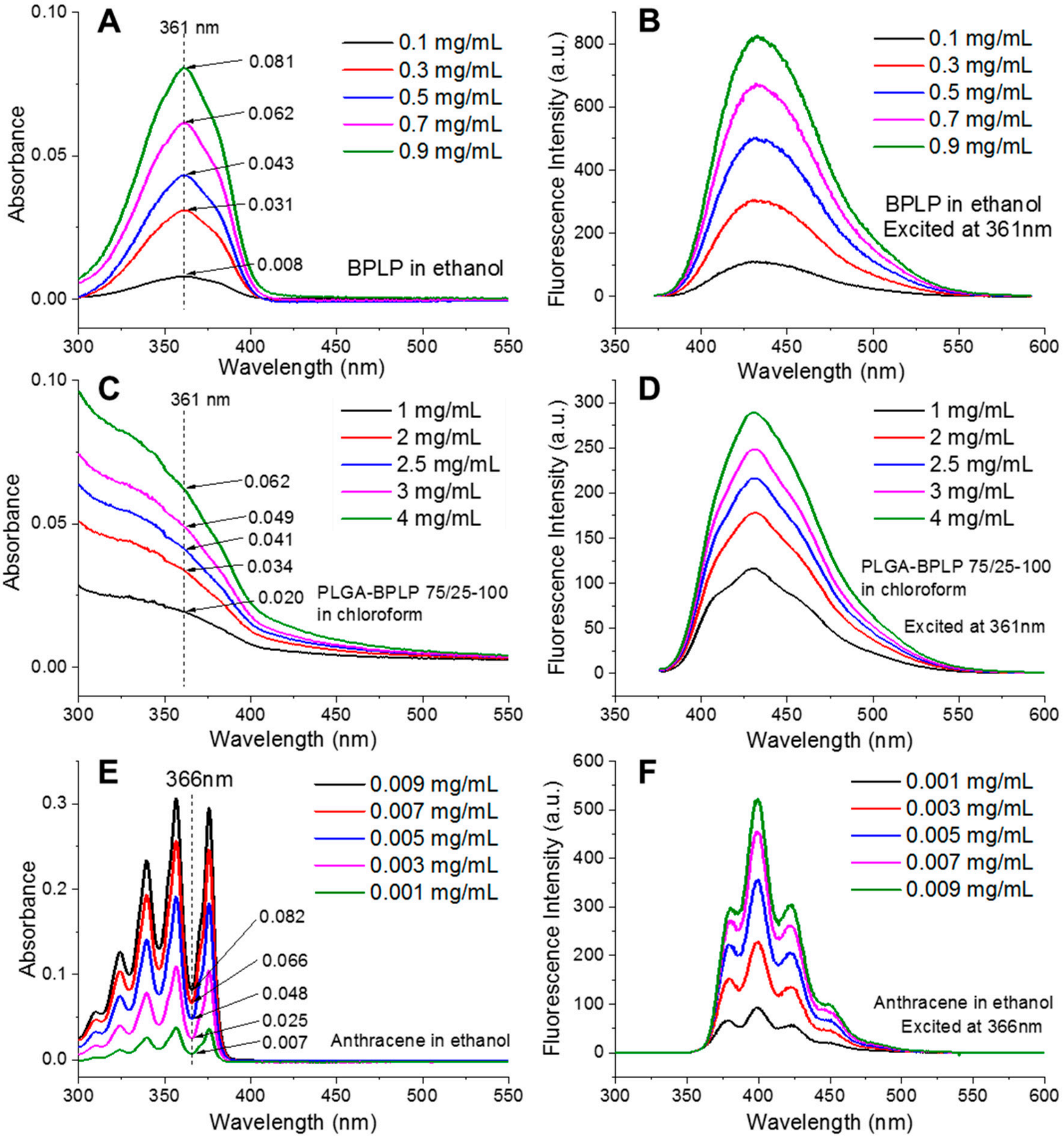

Figure S1. UV-Vis absorbance spectra and fluorescence spectra of the as-synthesized A/B) BPLP, C/D) PLGA-BPLP and E/F) reference anthracene at a series concentrations for fluorescence quantum yield calculation.

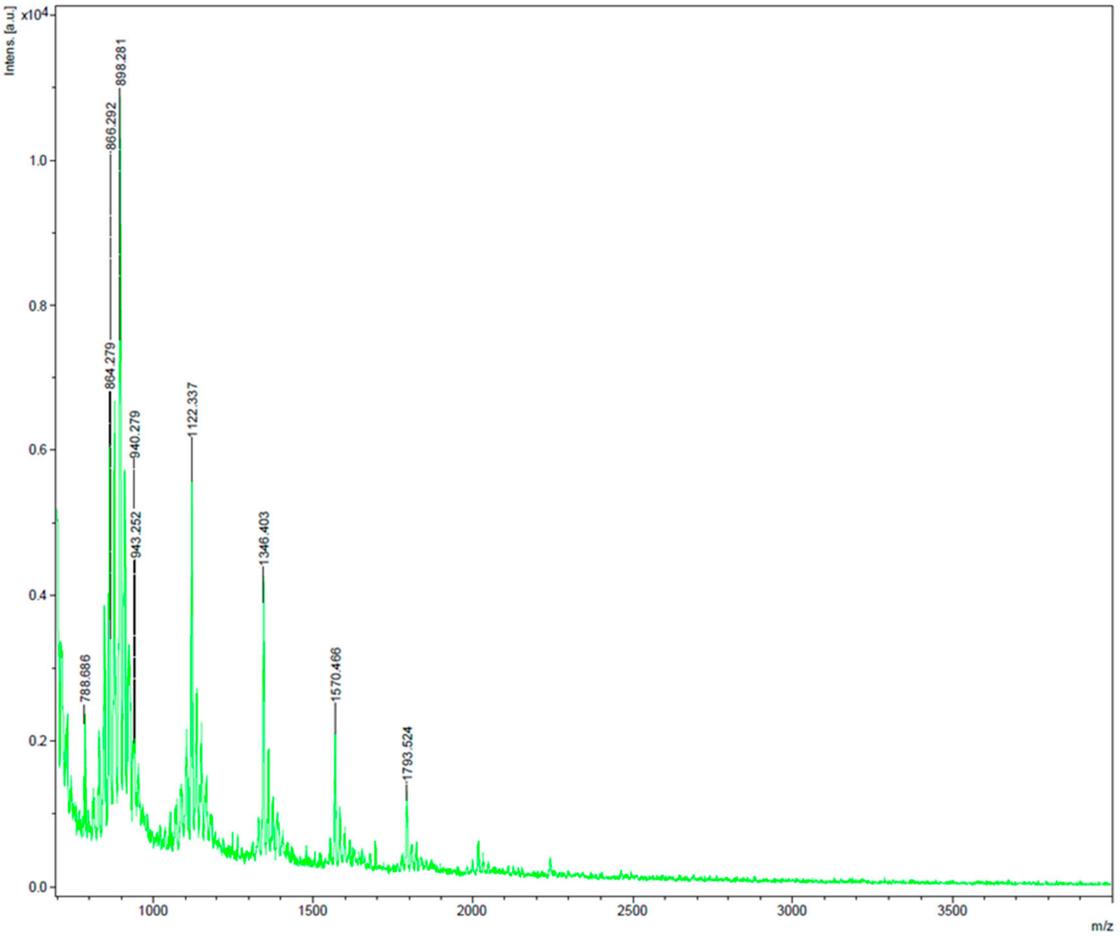

Figure S2. Matrix-assisted laser desorption/ionization time of flight mass spectroscopy (MALDI-TOF-MS) was used to determine the average molecular weight (M_w) of BPLP. Average M_w calculated from the mass spectrum of BPLP is 1044 g/mol. For the analysis, the sample was spotted in a MALDI plate with 1, 8, 9-Antracenotriol (Ditranol) and analyzed using a mass spectrometer (Bruker Daltonics Ultraflex TOF/TOF) in reflectron mode.

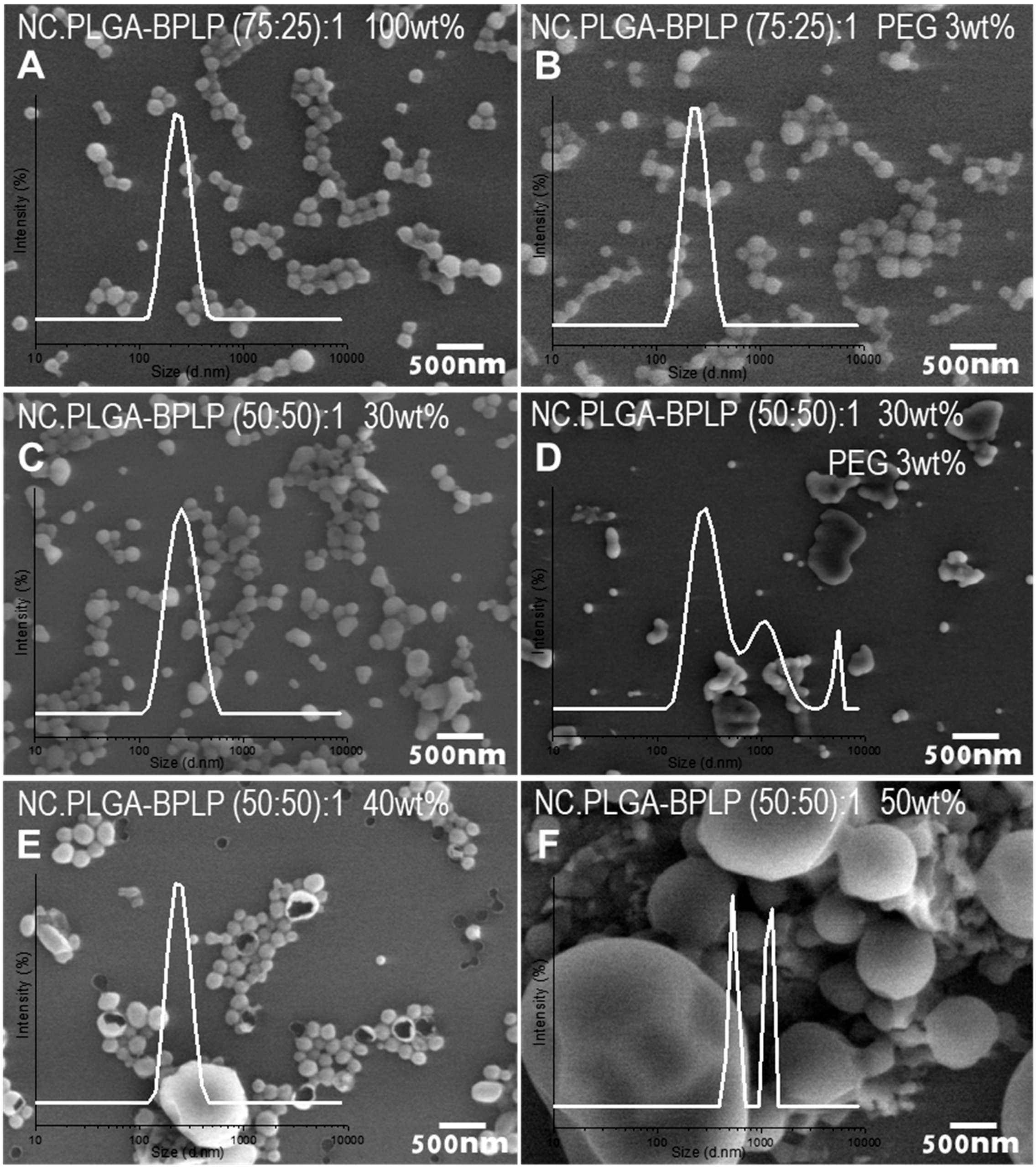

Figure S3. SEM images and DLS size distributions of NCs fabricated by different types of PLGA-BPLP.

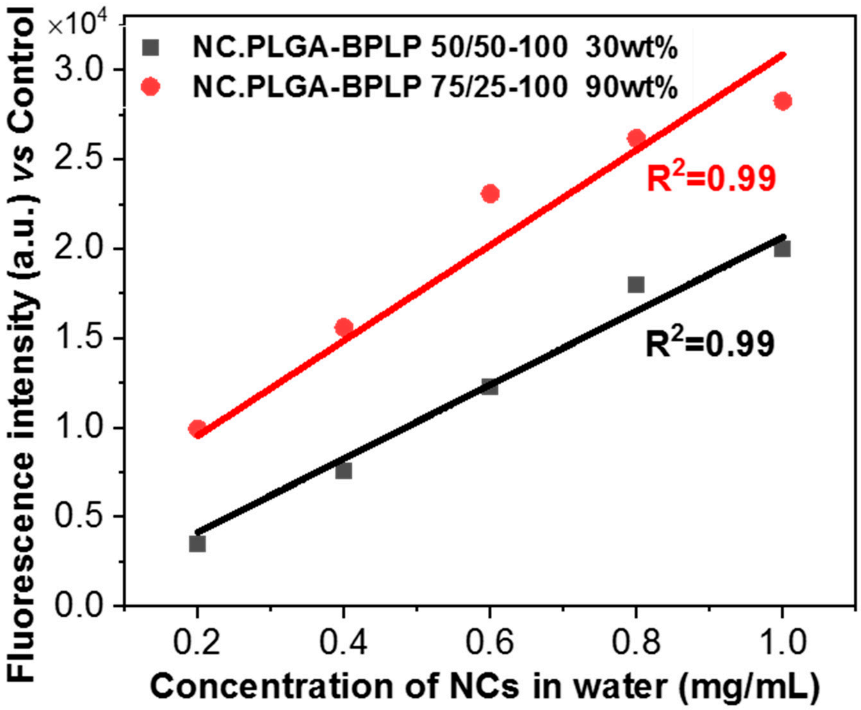

Figure S4. Fluorescence intensity of different concentrations of NCs measured by microplate reader at the maximal emission wavelengths (values have subtracted values of control non-fluorescence NCs).

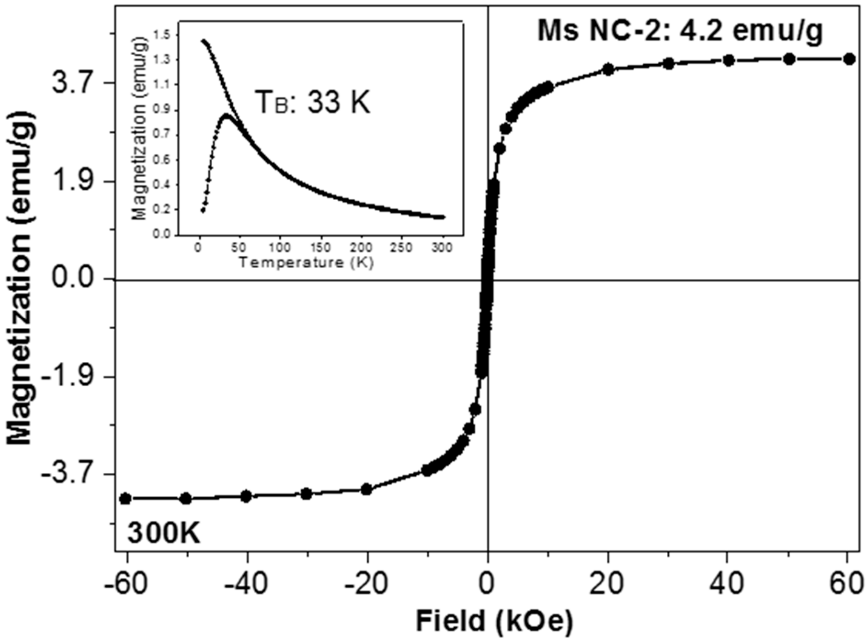

Figure S5. Hysteresis loop (300 K) and ZFC-FC (inset) measurements of the lyophilized nanocapsule batch NC2.
